# Patients With Epileptic Seizures and Multiple Sclerosis in a Multiple Sclerosis Center in Southern Germany Between 2003–2015

**DOI:** 10.3389/fneur.2019.00613

**Published:** 2019-06-06

**Authors:** Anja Schorner, Robert Weissert

**Affiliations:** Department of Neurology, University of Regensburg, Regensburg, Germany

**Keywords:** multiple sclerosis, epilepsy, seizure, incidence, prevalence

## Abstract

**Background:** So far, many studies have shown that the risk of developing seizures and epilepsy is higher among patients with multiple sclerosis (MS) than in the general population. However, the causal link between these two diseases is still unclear. In addition, it is not clearly understood whether and to what extent the manifestation of seizures and epilepsy in patients with MS affects the clinical course and the long-term prognosis of the disease. We aimed to retrospectively identify and describe patients with MS and with seizures and epilepsy which were seen at the Department of Neurology of the University of Regensburg in Germany between the years 2003–2015.

**Methods:** With the help of the electronic documentation system of hospital admitted patients followed by scrutinizing medical records of patients with MS for evidence of seizures and epilepsy, we identified patients with MS and seizures or epilepsy.

**Results:** We identified 22 individuals (1.74%) out of 1,267 patients with MS with seizures or epilepsy. 18 of these 22 individuals met criteria for epilepsy (1.42%). Nine MS patients (40.9%) suffered from relapsing-remitting MS (RRMS) whereas 11 MS patients (50.0%) showed a secondary progressive disease course (SPMS). Five of those (45.5%) converted from RRMS to SPMS before they acquired epilepsy. None of the identified patients with MS and seizures or epilepsy suffered from primary progressive MS (PPMS). Moreover, two MS patients (9.1%) had a history of seizures before MS onset. Seizures were of focal onset in 17 patients with MS (77.3%). Fourteen out of these 17 MS patients presented with focal to bilateral tonic-clonic seizures (82.4%). Five MS patients (22.7%) showed tonic clonic seizures of unknown onset. Status epilepticus was reported in three patients with MS (13%), for one patient the data was inconclusive.

**Conclusion:** The occurrence of seizures and epilepsy was higher than in the general population, suggesting a causal relationship between both diseases. In most cases, seizures occurred after the first manifestation of MS. The high frequency of focal seizures supports the concept of cerebral lesions in patients with MS playing an important role in precipitation of seizures and epilepsy.

## Introduction

Multiple sclerosis (MS) is a chronic inflammatory disease of the central nervous system (CNS) (1) which predominantly affects young adults, often female (2), and is of autoimmune origin (3). Because of the high variability in its clinical presentation, MS shows a wide range of different signs and symptoms (4). The appearance of seizures has previously been recognized as part of the MS disease spectrum (5). Although seizures are a rare manifestation in patients with MS, several studies indicate that the risk of developing seizures and epilepsy is three to six times higher in patients with MS compared to the general population (6–9). However, the underlying cause of the simultaneous occurrence of both diseases could not be described so far (4), and the pathophysiological mechanisms explaining this relationship are subject of current research (10). In addition, it is not clearly understood whether and to what extent the manifestation of seizures and epilepsy in MS patients affects the clinical course and the long-term prognosis (7, 9, 11).

The aim of this study was to identify and describe the patients who presented with MS and with seizures and epilepsy of the Department of Neurology at the University of Regensburg in Germany between 2003 and 2015. A comprehensive overview as well as a detailed characterization of this group of patients has been generated to allow the investigation of a potential contribution of inflammation and evolution of the lesions driving MS pathogenesis and progression to the manifestation of seizures and epilepsy.

## Subjects and Methods

The study was approved by the Ethics Committee of the University of Regensburg, Germany (Approval number: 16-101-0069). The period in which the analyzed data was collected ranged from April 15, 2016 to July 15, 2016. We identified 23 individuals out of 1267 patients with MS which simultaneously suffered from MS and seizures or epilepsy and presented themselves at the Department of Neurology of the University of Regensburg in Germany between 2003 and 2015. All patients with MS of the Department of Neurology of the University of Regensburg in this period are captured in this study and therefore there is no potential selection bias. As it is a retrospective study, the identification of the individuals was done with the help of the electronic admission system of the hospital and a computer search query, as well as by scrutinizing medical records of the MS patients for evidence of seizures and epilepsy within the individual archives of the hospital. Every single patient of the clinic is well documented in electronic and non-electronic form, so there is no anonymised data. The ICD numbers for MS–ICD-10: G.35–and for epilepsy–ICD-10: G.40–were used as search criteria for electronic search. In the following, the dates of birth of individual persons could be determined by archived physician letters and thus the associated files could be analyzed. No patients with clinically isolated syndromes (CIS) or pseudo tumoral presentation of MS were found in the records. As diagnostic criteria for MS the Poser criteria were used for all patients with first diagnosis of MS before 2001 and the McDonald criteria for patients with first diagnosis of MS after 2001. The Poser criteria differentiated between clinically definite MS, laboratory-supported definite MS, clinically probable MS and laboratory-supported probable MS based on clinical and paraclinical findings. Due to their early publication in 1983, they do not contain any MRI criteria for diagnosis (12). MRI findings are used with the McDonald criteria which have been revised several times within the last years (13).

Criteria of the International League Against Epilepsy (ILAE) of 2017 by Scheffer et al. were used to define epilepsy (14). Seizures were classified accordingly to Fisher et al. and arranged in seizures of focal, generalized, and unknown onset (15). Time zero for epilepsy was taken as the appearance of the first seizure.

Subsequently, the medical records of the patients with MS and seizures or epilepsy were collected and retrospectively reviewed. On this occasion the data and medical history of the individuals were evaluated in tabular and graphic form. Microsoft Office Word and Excel as well as the statistic program SPSS (version 23.0) were used. Thus, a detailed characterization of this cohort of MS patients and a description of the individual disease courses could be performed. The review of the individual patient records also revealed that some data were missing or incomplete, partly because some of the patients did not come to follow up visits after the first disease manifestation. As a result, the disease course of some patients could not be fully or completely reviewed.

## Results

### Number of Cases

We identified 23 individuals (1.82%) out of 1,267 patients with MS which suffered from seizures or epilepsy and presented themselves at the Department of Neurology of the University of Regensburg between the years 2003 and 2015. One out of 23 patients was excluded from the analysis because of intravenous drug abuse already in childhood. Accordingly, 22 MS patients (1.74%) which have had seizures or epilepsy remained. Eighteen of these 22 individuals met criteria for epilepsy (1.42%) (14), the other four persons had only exhibited one single seizure at time of data collection. Three of those patients had their first seizure at the time of latest presentation to the Department of Neurology of the University of Regensburg, one patient developed the first seizure a few years prior to the most recent follow up after sudden discontinuation of long-term medication with carbamazepine during an acute relapse of MS. The available data did not allow comparing the epilepsy incidence between patients with McDonald and Poser criteria, as all MS patients of the Department of Neurology of the University of Regensburg would have needed to be classified regarding these two criteria.

### Characteristics of Cases With Regard to MS

The mean age at the time of data collection was 48.5 ± 15.2 years (range 18–76 years) and most patients were females (17 females, 77.3%; 5 males, 22.7%). The mean age at the onset of the first symptoms of MS was 25.3 ± 8.7 years (range 14–42 years). Nine out of 22 patients (40.9%) suffered from relapsing-remitting MS (RRMS), whereas 11 patients (50.0%) showed a secondary progression of their disease (SPMS). Two patients (9.1%) could not be assigned to any MS type because the disease courses were unspecified in the medical records. There was no reported case of primary progressive MS (PPMS) in the investigated patient population. The mean duration of MS at the time of data collection was 24.87 ± 10.3 years (range 6–40 years). The most common symptoms at the first onset of the MS disease manifestation were sensory disturbances followed by visual impairment.

Ten of the 22 patients with MS (45.5%) were treated with interferon-beta at any time during their disease course. Other immunomodulatory medications used for treatment of MS included azathioprine (*n* = 6), mitoxantrone (*n* = 5), glatiramer acetate (*n* = 4), natalizumab (*n* = 4), and fingolimod (*n* = 3); baclofen was used for symptomatic treatment of spasticity (*n* = 5).

### Manifestation of Seizures in the Disease Course of MS

Two patients (9.1%) had a history of seizures before MS onset and 16 patients (72.7%) had experienced seizures after the onset of the disease. The first appearance of seizures for the remaining four patients (18.2%) could not be clarified by the assessment of their medical records. Nine patients with MS (40.9%) had RRMS at the time of first seizure.

Eleven patients developed SPMS (50.0%). Five of those patients (45.5%) converted from RRMS to SPMS before and four patients (36.4%) after they developed seizures. The medical records of the remaining two patients (18.2%) did not contain enough information to draw a conclusion on the transition point to SPMS.

### Characteristics of Seizures and Treatment

Seizures were of focal onset in 17 patients (77.3%). Fourteen out of these 17 patients presented with focal to bilateral tonic-clonic seizures (82.4%). Furthermore, five patients (22.7%) showed tonic-clonic seizures of unknown onset. Three of those were diagnosed with unknown epilepsy; two were not seen any more in the hospital after their first seizure. Fourteen patients developed bilateral tonic-clonic seizures (63.6%) after initial presentation with focal seizures; five patients suffered from tonic-clonic seizures of unknown onset (22.7%). Therefore, a total of 19 patients suffered from seizures involving both hemispheres (86.4%). One of the five patients with tonic-clonic seizures of unknown onset showed hippocampal atrophy in NMR imaging studies. In one of these patients an ictal EEG with event-related potentials was recorded, in three other patients only interictal EEGs were recorded. Two of those showed abnormalities such as focal slowing or dysrhythmia. In one patient no EEG recordings were performed. Altogether, three of these five patients with tonic-clonic seizures of unknown onset were classified as having an unknown epilepsy type. Status epilepticus was reported in three patients (13.6%), for one patient the data was inconclusive. In two patients with MS and seizures (9.1%) other factors were identified which may have contributed to the occurrence of seizures namely dehydration and hypokalaemia as well as alcohol abuse. However, after careful consideration and detailed assessment of the patient files, these two patients were not excluded from the analysis.

The frequency and the severity of the seizures varied from patient to patient. In the assessed time window in 21 individuals (95.7%) at least one antiepileptic drug therapy was documented at the time point of data collection. The most commonly used antiepileptic drugs included levetiracetam, lamotrigine, carbamazepine, and valproate. Less used medication contained lorazepam, gabapentin, phenytoin, and lacosamide. Both mono- and polytherapies were prescribed, with nine patients requiring only one drug to treat their seizures. All other patients received at least two different medications during the assessment period. Only one patient did not receive any antiepileptic drug. Some patients had an excellent response to anticonvulsive therapy, whereas the epilepsy of the other part of the group of patients seemed to be difficult to control and was characterized by recurrent seizures of varying frequencies. In seven patients the emergence of seizures could be controlled. The other seven patients seemed to suffer from drug-resistant seizures. One more patient developed recurrent seizures probably because of non-compliant drug use. Five patients had their first seizure and did not present to the clinic again prior to the time of data collection, so it remains unclear whether these patients developed more seizures under antiepileptic therapy or not. Documentation was incomplete for two of the patients and therefore no statement regarding therapy response can be made.

Unfortunately, it was difficult to compare epilepsy outcome based on the use of immunomodulatory or symptomatic MS treatment due to irregular presentation of some patients at the Department of Neurology of the University of Regensburg resulting in insufficient information being available on this subject. Nevertheless, an increased frequency of seizures could be determined in two individuals whose medication was changed to fingolimod.

The characteristics of the 22 described individuals are presented in Table 1.

**Table 1:** Overview of the MS patients with epileptic seizures at the Department of Neurology of the University of Regensburg which have presented themselves between 2003 and 2015.

	Age range^*^	**Age (years)** **fm MS**	**Age (years)** **fd MS**	**Disease** **course** **of MS**	**Disease** **state** **of MS^**a**^**	**Duration of MS (years)^**a**^**	**Age (years)** **fm EP**	**Age** **(years)** **fd EP**	**FS**	**FBTCS**	**TCSUO**	**Status epilepticus**	**MS** **before** **EP**	**EP** **before** **MS**	**IFN-** **beta^**a**^**	**Drug resistant epilepsy**
	45-60	38	40	RRMS	RRMS	2	40	40	+	+	–	–	+	–	–	–
	18–30	15	15	RRMS	RRMS	6	21	21	–	–	+	–	+	–	+	–
	45–60	14	35	RRMS → SPMS	SPMS	21	34	35	+	+	–	+	+	–	–	+
	31–45	14	16	RRMS	RRMS	15	28	29	+	–	–	–	+	–	+	+
	18–30	×	18	RRMS	RRMS	×	23	23	+	+	–	–	+	–	–	–
	45–60	19	21	RRMS → SPMS	SPMS	30	49	49	+	+	–	–	+	–	–	–
	45–60	28	28	RRMS → SPMS	SPMS	22	50	50	+	+	–	–	+	–	+	–
	60–80	×	×	RRMS → SPMS	SPMS	×	60	60	–	–	+	–	+	–	–	×
	45–60	×	29	RRMS	RRMS	×	45	45	–	–	+	+	+	–	+	+
	60–80	33	36	RRMS → SPMS	×	22	55	55	–	–	+	+	+	–	–	×
	45–60	26	26	RRMS → SPMS	SPMS	26	52	52	+	+	–	+	+	–	–	–
	31–45	20	20	RRMS → SPMS	RRMS	12	32	32	+	+	–	–	+	–	+	–
	45–60	25	25	RRMS → SPMS	RRMS	16	×	41	+	+	–	–	+	–	+	–
	60–80	42	62	×	×	19	59	61	+	–	–	–	+	–	–	×
	18–30	×	12	RRMS	RRMS	×	12	12	+	+	–	–	+	–	+	–
	60–80	×	39	×	×	×	×	×	+	+	–	–	×	×	+	+
	31–45	29	30	RRMS	×	×	15	16	+	+	–	–	–	+	–	–
	45–60	34	44	RRMS	×	0	33	34	+	+	–	–	–	+	+	+
	60–80	×	32	RRMS → SPMS	×	×	×	×	+	–	–	–	×	×	–	×
	31–45	×	20	RRMS → SPMS	×	×	×	12	+	+	–	–	×	×	–	+
	31–45	21	24	RRMS	RRMS	8	29	29	+	+	–	–	+	–	+	+
	31–45	21	24	RRMS → SPMS	×	×	×	×	–	–	+	–	×	×	–	–
Mean	47.7	24.9	28.0			9.0	37.5	36.6								
Range	18 – 76	14 – 42	12 – 62			0 – 30	12 – 60	12 – 61								

*In order to protect the identity of the individuals and since it is a requirement of the journal, age ranges were used instead of the exact ages of the patients*.

* *At data collection*.

a *At time of epilepsy diagnosis*.

*fm, first manifestation; fd, first diagnosis; FS, Seizures of focal onset includes FAS (focal aware seizures) and FAIS (focal impaired awareness seizures); FBTCS, focal to bilateral tonic-clonic seizures; TCSUO, tonic-clonic seizures of unknown onset; EP, epilepsy; IFN, interferon; x, unknown*.

b *Therapy with interferon-beta at any time during the disease course of MS*.

## Discussion

Even though seizures only affect a minority of patients with MS, they are still a serious problem (5). The occurrence of seizures has previously been considered as part of the disease spectrum of MS. Several studies indicate that the risk of developing seizures is up to six times higher in MS patients compared to the general population (6–9).

It is well-known that MS is not only a disease of the white matter of the cerebral cortex, but also affects the gray matter (16, 17) and that atrophy and lesions in this part of the brain are more frequent than previously suggested (4). This leads to the assumption that cortical and subcortical lesions as well as surrounding oedema in MS may play an epileptogenic role. An increased number of these lesions in MS patients with comorbid seizures and epilepsy has been reported as compared to MS patients without seizures (5, 12, 18). NMR-tomographic studies have shown an association between gray matter lesions and the appearance of epilepsy (7, 19–21). A recent study of Burman et al. demonstrated an association between epilepsy and a progressive disease course, possibly due to gray matter lesions (22, 23). The increased lesion load possibly results in a demyelinated area with a higher risk of epileptogenic potential (24). Furthermore, some medications used in treating MS, such as baclofen or interferon beta, may increase the risk of generating seizures. The proconvulsive properties of some drugs could be due to metabolic interference with antiepileptic drugs or due to direct neurotoxic effects (25, 26).

While the prevalence of epilepsy in the general population is about 0.27–1.7%, it seems to be three to six times higher in MS patients (27). Based on several studies by different authors, prevalence rates of seizures or epilepsy between 0.5 and 8.3% have been reported (5, 27). We observed a prevalence for seizures of 1.74% and for epilepsy of 1.42% in our MS cohort, because only 18 of the 22 patients met criteria for epilepsy while the other four patients presented only one seizure. Accordingly, the frequencies reported by us are lower than the frequency of people having MS and seizures or epilepsy previously reported in other studies as shown in Table 2.

**Table 2:** MS patients observed in other studies of different MS centres and countries and the amount of concomitant seizures in these patients.

**References**	**Patients with** **MS (n)**	**Patients with MS** **and EP (*n*)**	**Patients with MS and EP (%)**	**Country**	**Diagnostic criteria** **for MS**
Benjaminsen et al. (25)	431	19	4.4	Norway	Poser/McDonald
Burman et al. (22)	14545	502	3.5	Sweden	-
Catenoix et al. (28)	5041	67	1.3	France	Poser
Eriksson et al. (29)	255	20	7.84	Sweden	Poser
Etemadifar et al. (30)	3522	81	2.3	Iran	McDonald
Lund et al. (31)	364	24	6.6	Norway	Poser
Moreau et al. (7)	402	17	4.25	France	Poser
Olaffson et al. (9)	188	5	2.65	Iceland	Poser
Shaygannejad et al. (32)	920	29	3.15	Iran	McDonald
Sokic et al. (20)	268	20	7.5	Serbia	Poser
Striano et al. (33)	270	13	4.8	Italy	McDonald
Uribe-San-Martín et al. (10)	310	10	3.2	Chile	McDonald
Viveiros et al. (34)	160	5	2.5	Brasil	McDonald
Zare et al. (35)	920	29	3.15	Iran	McDonald

Such varying results could be due to selection bias and differences in methods, definitions and diagnostic criteria (30, 34). For example, the accuracy of the MS diagnosis as well as the definition of epilepsy and its distinction toward non-epileptogenic, paroxysmal manifestations of MS, such as tonic spasm of the extremities, play a crucial role (36). Moreover, the different composition of the study populations may have also contributed to the varying results. Some of the listed studies might have included all MS patients with seizures independent of these having other reasons for developing seizures than MS. For example, Catenoix et al. reported a prevalence rate of 2.0% for MS patients with seizures whereas the prevalence rate was less (1.3%) when excluding all patients that might have had other reasons for seizures than MS (28). It has also to be noted that some studies might have included MS patients who developed their first seizure long before MS onset, making a relation between the two diseases rather unlikely (37). Furthermore, it should be emphasized that no strict diagnostic criteria for MS existed before 1983 and that magnetic resonance imaging (MRI) was first introduced in the diagnostic criteria of MS in the mid-1980s (12). It cannot be excluded that the prevalence rate may have been underestimated because of the retrospective design of our study.

With a percentage of 77.3 %, women were significantly overrepresented in our study population. The same gender preference was shown in other studies by various authors (6, 7, 27, 35, 37), suggesting an increased occurrence of epilepsy especially in women with MS. In addition, in this study, patients presented with an average age of 25.3 years at the onset of MS. Other studies too reported their patients being relatively young when MS was diagnosed for the first time (10, 38). This in turn raises the suspicion that the risk of developing epilepsy is highest among the younger MS patients (10, 38). However, as MS is generally more likely to affect younger people and among these predominantly women, these numbers probably reflect the distribution in the general MS population (2, 6, 35).

The most common symptoms at the first onset of the MS disease manifestation were sensory disturbances followed by visual impairment. Symptoms of these functional systems are among others the most common disease manifestations in MS patients (39).

Considering the time of first manifestation of seizures and epilepsy in MS patients, it must be noted that seizures may occur in any subtype of MS and at any time during the disease course. Furthermore, the appearance of epileptic symptoms is possible even before the onset of MS. However, it should be emphasized that MS itself can exist long before becoming clinically apparent for the first time (5, 11, 27). The predominant MS subtypes in our study were RRMS and SPMS. No PPMS patients were present in our cohort but PPMS patients have been reported in other studies in which the presentation of MS and epilepsy has been investigated (6, 10, 21, 22, 25, 26, 28, 30, 40).

As already mentioned, focal pathologies of the brain occurring in MS patients, which are likely supposed to cause increased excitability of the cerebral cortex, might be the underlying cause of seizures in these patients (7, 20, 25). The lower frequency of seizures in patients with PPMS may be linked to the fact that these patients show a lower burden of cerebral lesions, predominantly in form of non-periventricular distribution (23).

Various studies have shown that epilepsy usually occurs in early stages of MS (11). Seizures may be the first symptom of MS, however most seizures occur during the disease and thus after diagnosis of MS (32). The present study confirms that the majority of seizures occur in patients already diagnosed with MS. Seizures occurred about 14.92 years (range one to 30 years) after the first manifestation of MS and thus much later as compared to previously published numbers (7, 20, 28, 32, 35). However, some studies also reported a larger range between the first manifestation of MS and occurrence of epilepsy which are in line with the range presented in the current study (25, 33, 40). These differences may be explained by the fact that some of these studies are older and were published at a time when uniform diagnostic criteria for MS were not yet available and diagnostic options (e.g., MRI) were limited. Consequently, this may have led to a later diagnosis of MS and a shorter duration until the first onset of epilepsy. In addition, the patients' better understanding of their disease, their education, the use of self-help groups and a resulting more conscientious and cautious lifestyle as well as progress in therapy, especially immunotherapy, may cause a later appearance of seizures in MS patients (39). Since there was no patient in this study who showed seizures as the first symptom, it is suggested that the likelihood of developing seizures and epilepsy may rise with the duration of MS and the number of lesions (19).

Some authors mention that, although all types of seizures may occur in MS, seizures of focal onset are more common than tonic-clonic seizures of unknown onset, with a high proportion of focal to bilateral tonic-clonic seizures among seizures of focal onset (7, 18, 27, 34). This is in line with the results of our study, in which 77.3% of affected individuals had seizures of focal onset of which 82.4% developed into focal to bilateral tonic-clonic seizures. Only 22.7% initially had tonic-clonic seizures of unknown onset. This supports the assumption that local inflammation and lesion evolution in the brain could be the cause of developing epilepsy when already having MS (10, 25). Three of the five patients with tonic-clonic seizures of unknown onset were classified as having unknown epilepsy. No patients with a proven generalized epilepsy were identified (14).

Whether and to what extent the manifestation of seizures and epilepsy in MS patients influences the clinical course and the long-term prognosis of the MS has not been sufficiently clarified (7, 9, 11). Patients with MS and simultaneously existing seizures or epilepsy show a higher EDSS score as compared to MS patients without epileptic manifestations (18, 28, 41). In addition, seizures are associated with the earlier loss of walking ability and, consequently, use of a wheelchair, as well as earlier death (26).

When studying the patients' medical records, it was apparent that seizures differed in both severity and frequency, so that some patients where more affected than others by their disease. In addition, it could be seen that epilepsy in some patients was halted when using anticonvulsive therapy while the epilepsy of the other patients seemed to be drug resistant and difficult to control by medical therapies, so these patients presented recurring seizures. Similar findings were reported in a study by Dagiasi et al. in which the number of seizure free patients was rather low (42).

In the literature, two different patterns of epilepsy are distinguished in the case of simultaneously existing MS: the so-called “benign” and “progressive” course of epilepsy. The benign form contains an unchanged or even decreasing frequency of seizures and a regular pattern of their appearance. The progressive course, however, describes a frequent occurrence of seizures and a constant development of new types of seizures (37).

In our study, 50.0% of patients with MS suffered from SPMS. Five patients had their first seizure after developing SPMS, which leads to the assumption that the increased load of cerebral lesions occurring in MS patients may act as a risk factor for the development of epilepsy. Four patients suffered from seizures before RRMS transformed to SPMS. This on the other hand suggests that epilepsy as a comorbidity of MS could lead to a higher severity of the disease, to an increasing disability and to an earlier development of SPMS.

While some authors believe that seizures in MS are usually harmless and show an adequate response to antiepileptic therapy, Engelsen et al. in contrary, have the opinion that there is a poorer prognosis of treatment efficacy of seizures among MS patients (6). Possibly there is a higher risk for developing status epilepticus including all its serious consequences in patients with MS. As described above, in our study, status epilepticus was confirmed in three individuals, whereas for one patient the data were inconclusive.

It is possible that MS patients with concomitant seizures experience a more severe disease course as MS patients without seizures. Thus, seizures could probably be considered as an additional factor aggravating disease. This might be because seizures can affect disability itself and lead to increased neuronal damage with the resulting consequences (43).

Whether the appearance of both disorders is coincidental or MS acts as a non-specific trigger for developing seizures and epilepsy or indeed is the direct cause of seizures is still under debate (28). However, the increased incidence of epileptogenic activities in MS patients as compared to the non-MS population suggests a causal relationship between the two diseases (26).

## Conclusion

The findings of this study indicate that there is a higher risk of developing seizures when having MS as compared to the general population. The fact that in most cases, seizures occurred after the first manifestation of MS indicates that they may be the consequence of MS. Furthermore, a relation between certain immunomodulatory therapies and the development and frequency of seizures in MS patients seems possible but remains to be proven in further studies due to limited data availability at present. Moreover, the high frequency of RRMS and SPMS as well as of focal epilepsy supports the idea that cortical lesions occurring in MS patients may play an important role in comorbid epilepsy. Some patients had their seizures before developing SPMS while seizures in others appeared after evolving SPMS. This may indicate that epilepsy is a marker of more aggressive forms of MS but could also suggest that progressive forms of MS are more likely to generate seizures.

## Ethics Statement

The study was approved by the Ethics Committee of the University of Regensburg, Germany (Approval number: 16-101-0069).

## Author Contributions

RW outlined the subject of the research theme. RW and AS obtained ethical permission to perform the research. AS and RW searched the patient files and collected the original data. AS analyzed the data. AS and RW interpreted literature and wrote the manuscript. AS and RW agreed to be accountable for all aspects of the work.

### Conflict of Interest Statement

The authors declare that the research was conducted in the absence of any commercial or financial relationships that could be construed as a potential conflict of interest.
